# Micellization of Pluronic P123 in Water/Ethanol/Turpentine Oil Mixed Solvents: Hybrid Particle–Field Molecular Dynamic Simulation

**DOI:** 10.3390/polym11111806

**Published:** 2019-11-03

**Authors:** Ying Zhao, Su-Min Ma, Bin Li, Antonio De Nicola, Nai-Sen Yu, Bin Dong

**Affiliations:** 1 Institute of Nano-photonics, School of Physics and Materials Engineering, Dalian Minzu University, Dalian 116600, Chinayunaisen@dlnu.edu.cn (N.-S.Y.); 2 Institute of Theoretical Chemistry, Jilin University, Changchun 130021, China; 3 School of Chemical Engineering and Technology, Sun Yat-sen University, Zhuhai 519082, China; libin76@mail.sysu.edu.cn; 4 Department of Organic Materials Science, Yamagata University, 4-3-16 Jonan, Yonezawa, Yamagata-ken 992-8510, Japan; adenicola@yz.yamagata-u.ac.jp

**Keywords:** hybrid particle–field molecular dynamics, block copolymer, self-assembly, P123, ethanol, turpentine oil

## Abstract

The hybrid particle–field molecular dynamics simulation method (MD-SCF) was applied to study the self-assembly of Pluronic PEO_20_-PPO_70_-PEO_20_ (P123) in water/ethanol/turpentine oil- mixed solvents. In particular, the micellization process of P123 at low concentration (less than 20%) in water/ethanol/turpentine oil-mixed solvents was investigated. The aggregation number, radius of gyration, and radial density profiles were calculated and compared with experimental data to characterize the structures of the micelles self-assembled from P123 in the mixed solvent. This study confirms that the larger-sized micelles are formed in the presence of ethanol, in addition to the turpentine oil-swollen micelles. Furthermore, the spherical micelles and vesicles were both observed in the self-assembly of P123 in the water/ethanol/turpentine oil-mixed solvent. The results of this work aid the understanding of the influence of ethanol and oil on P123 micellization, which will help with the design of effective copolymer-based formulations.

## 1. Introduction

The self-assembly of amphiphilic block copolymers has attracted considerable attention for many decades because they can form many novel structures, including spheres, cylinders, bicontinuous phases, lamellae, vesicles, and many other complex or hierarchical assemblies [1,2,3,4]. Amphiphilic block copolymers have been widely used in many industrial applications, such as detergents, dispersion stabilizers, foaming agents, emulsifiers, and lubricants [5]. Triblock copolymers composed of hydrophilic (polyethylene oxide)*_m_* −amphiphilic (polypropylene oxide)*_n_*− hydrophilic (polyethylene oxide)*_m_* blocks are commercially known as Pluronics or Poloxamer [6]. By changing the relative composition of the polyethylene oxide (PEO) and polypropylene oxide (PPO) blocks (general formula: (PEO)_m_−(PPO)_n_−(PEO)_m_), it is possible to tune the hydrophilic–lipophilic balance (HLB), which makes the main structural differences between Pluronics. Since the mid-1960s, Pluronics have been widely and successfully used in biomedical applications, due to their biocompatibility and non-toxicity, for example, micellar nanocarriers for drug delivery, biological reaction modifiers in chemistry, non-viral gene therapy agents, and wound dressings for the treatment of thermal burns [7,8,9,10,11,12,13]. Pluronics P123 (PEO_20_-PPO_70_-PEO_20_) is one of the most used block-copolymers for several applications. For these reasons, the micellar assembly of P123 in water has been widely studied by a variety of experimental techniques, such as static light scattering, fluorescence spectroscopy, and surface tension measurements [13,14,15,16]. Furthermore, the effect of mixed solvents on the aggregation of P123 [17] and the phase behavior of P123 in mixed ethanolic solvents has been the subject of several studies [18,19]. Very recently, Chat et al. successfully investigated the aggregation features of P123 in the presence of lavender oil and ethanol using dynamic light scattering, small angle neutron scattering, and rheology [20]. They showed that lavender oil assisted aggregation characteristics of Pluronic P123 in the mixed solvents of ethanol and water [20]. Furthermore, Ganguly et al. studied tea tree essential oil (TTO) on the properties of aqueous solutions of Pluronic P123 and the effect on micellar solubilization. They showed that the solubilization of TTO brings about spherical-to-worm-like micelles-to-vesicular structural transitions in aqueous solutions of Pluronic P123 [21]. 

Although experimental data can help with the understanding of the micellization mechanism of P123 in mixed solutions, it is hard to obtain good information of the phenomenon at molecular level. Meanwhile, using computer simulation methods to study the self-assembled structure and properties of block copolymers in aqueous solution has gradually attracted scientists’ attention [21,22,23,24]. Computer simulations can not only explain the underlying self-assembly mechanism, but also offer a route for designing and predicting future experiments. In particular, the standard all-atom molecular dynamics (AAMD) method cannot be directly performed to investigate the self-assembly phenomenon because of the time and length scale limits. Therefore, mesoscopic models, such as dissipative particle dynamics (DPD) [25,26,27,28,29,30], MesoDyn [31], and the Monte Carlo method [32,33], which have access to larger time and length scales, have successfully been used to investigate the micellization phenomenon. For example, Huang et al. used DPD to simulate the entire phase diagram of Pluronic L64 and water. Furthermore, they also proved that non-Newtonian behaviors can be predicted by non-equilibrium DPD simulation [25]. Recently, a different computational approach, the hybrid particle–field MD (MD-SCF) method, was developed, in which the MD and self-consistent field (SCF) method are combined [34,35]. This hybrid particle–field MD method not only has similar accessibility to large time and length scales of pure SCF methods, but also has a chemical specificity that is similar to the MD method. In particular, in our previous works, we presented coarse-grained (CG) hybrid particle–field models to study the phase behavior of the mixture of phospholipid-water [36], Triton TX-100 surfactant [37,38], Pluronic-water [39], and nanocomposite systems [40,41]. However, to the best of our knowledge, studying the effect of oil on the aggregation behavior of P123 in the mixed solvents using a computer simulation method is still lacking.

In this paper, we present a study of the aggregation features of Pluronic P123 in the presence of water, ethanol, and turpentine oil, using a MD-SCF simulation. This paper aims to understand the self-assembly mechanism of micellization of the P123 in the mixed solvents at the molecular level. Furthermore, the influence of turpentine oil on the aggregation behavior of P123 is also discussed. 

## 2. Materials and Methods

### 2.1. Simulation Method

In 2009, a computer simulation method of hybrid particle–field molecular dynamics method was developed [34,36,39]. The main feature of this method is to combine the molecular dynamics method based on particle description with the average field theory of self-consistent field. That is, the non-bonded interaction between particles of different molecules can be replaced by the evaluation for each particle of those with an external potential that depends on the local density at position *r*, and can solve the multi-body problem [35] using the molecular dynamics simulation method. In the framework of the SCF theory, one chain is regarded to interact with the surrounding chains not directly but through a mean field. Its calculation method is as follows: the Hamilton operator consists with two parts composed of *M* molecules, which can be expressed as:

(1) $$\hat{H}(\Gamma )={\hat{H}}_{0}(\Gamma )+\hat{W}(\Gamma )$$

${\hat{H}}_{0}(\Gamma )$ is the Hamiltonian of a reference ideal system composed of *M* molecules but with all the bonded and non-bonded interaction in the standard MD simulations. Γ specifies a point in the phase space, and the hat symbol ∧ indicates that the relevant physical quantity is a function of the microscopic states described by the phase space Γ. On the other hand, the deviation from the reference system, which is induced by non-bonded interactions between molecules, is accounted for by the term $\hat{W}(\Gamma )$.

Then, the total partition function of the system is written as:

(2) $$Z=\frac{1}{M!}\int d\Gamma exp\left\{-\beta \left[{\hat{H}}_{0}(\Gamma )+\hat{W}(\Gamma )\right]\right\}$$

where $\beta =\frac{1}{k_{B}T}$. 

From the microscopical level, the density distribution of particles is defined as the sum of δ functions centered at the center of mass of each particle as:

(3) $$\hat{\phi }(r;\Gamma )=\sum_{P=1}^{M}\sum_{i=0}^{S(p)}\delta (r-r_{i}{}^{\left(p\right)})$$

where $r_{i}{}^{\left(p\right)}$ is defined as the particles’ coordinate of the *P*th molecule, S(p) is defined as the number of particles contained in the *P*th molecule, and *M* is the total number of molecules in the system. We assume that the particle density of the phase space determines the external field:

(4) $$\hat{W}(\Gamma )=\hat{W}(\phi (r;\Gamma ))$$

Here, we assume that $\hat{W}(\phi (r;\Gamma ))$ depends on Γ only through the particle density ϕ(r;Γ). Then, we can write the single-molecule partition function as:

(5) $$Z=\frac{1}{M!}\int D\left\{\phi (r)\right\}\int D\left\{w(r)\right\}\times exp\left\{-\beta \left[-\frac{M}{\beta }\ln Z+W[\phi (r)]-\int V(r)\phi (r)dr\right]\right\}$$

In this expression, *Z* is partition function of the single molecule, w(r) is a conjugate field of ϕ(r), and V(r) is the external potential. 

Next, we assume that the density dependent interaction potential W, where each species is specified by the index K, takes the following form in Equation (6):

(6) $$W\left[\left\{\phi_{K}(r)\right\}\right]=\int dr\left(\frac{k_{B}T}{2}\sum_{{KK}^{\prime }}\chi_{{KK}^{\prime }}\phi_{K}(r)\phi_{K^{\prime }}(r)+\frac{1}{2\kappa }\left(\sum_{K}\phi_{K}(r)-\phi_{0}\right)\right)$$

where $\phi_{K}(r)$ is the coarse-grained density of the species K at position *r*, and $\chi_{{KK}^{\prime }}$ represents the mean field parameters for the interaction of a particle of type K with the density fields due to particles of type $K^{\prime }$. In particular, κ is the compressibility that is assumed to be sufficiently small to obtain the relaxed incompressibility condition. Then, the external potential of type K can be given by:

(7) $$V_{K}(r)=\frac{\delta W[(\phi_{K}(r)]}{\delta \phi_{K}(r)}=k_{B}T\sum_{K^{\prime }}\chi_{{KK}^{\prime }}\phi_{K^{\prime }}(r)+\frac{1}{\kappa }(\sum_{K}\phi_{K}(r)-\phi_{0})$$

Finally, the density derivatives of Equation (7) are required to calculate the forces, further details can be found in [34]. Moreover, the values of the coarse-grained density at lattice points are not updated at every time step but only at every prefixed density update time. Between two updates, the values of the densities on the lattice are used to interpolate density derivatives and, hence, forces. Details concerning the OCCAM code used to perform the MD-SCF simulations are reported in [35]. 

In the hybrid particle–field molecular dynamics simulations, the box length of the system was 64.25 nm. The total number of particles in the system was 192,000, and the diameter of the particle σ was set at 1.046 nm. The number density was 0.725. The time step used for the integration of the equations of motion was set to 0.03 ps. The grid size was set to 2.1 nm, and the density field was updated every 300 time steps (Update frequency has been tested in several of our previous papers and guarantees reasonable results with respect to finer methods [35,36,37,38,39,41].) The NVT ensemble was chosen, the temperature was kept constant at 303K, the same as in the experiments of Reference [20], using the Andersen thermostat with a collision frequency of 20 ps^−1^. The total simulation step was 8 × 10^7^ steps, which corresponds to the real time 2.4 µs. The detailed information of the simulation systems is reported in Table 1 and Table 2.

### 2.2. Coarse-Grained (CG) Model

**Figure 1:**
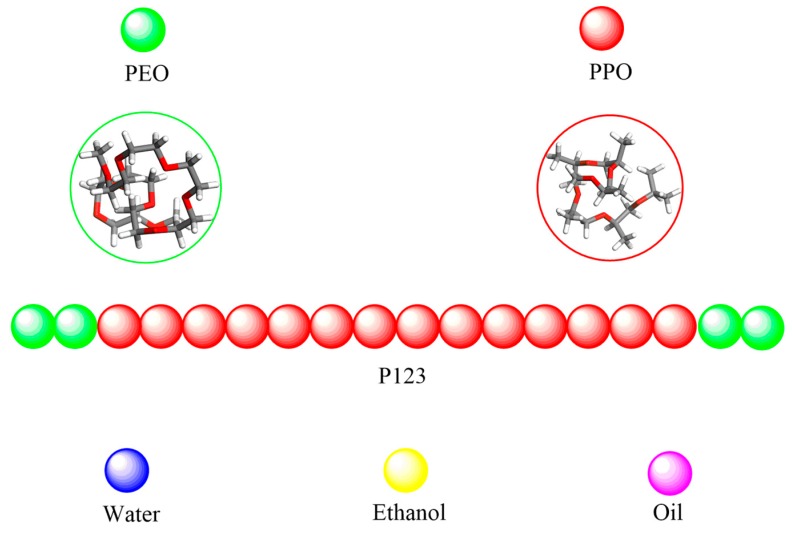
Mapping scheme of the coarse-grained models of P123 (EO2PO14EO2), water (corresponds to 20 H_2_O monomers), ethanol (corresponds to 6 ethanol monomers), turpentine oil (corresponds to 2 monomers).

The coarse-grained model was used in our simulations, in which the mapping scheme was similar to our previous work [29,30]. According to the molecular weight and experimental bulk densities, the monomer volumes were obtained. In particular, we took 20 water molecules and grouped them together into one bead with a volume of 600 Å^3^. The same bead volume corresponds approximately to 9 PEO monomers, 5 PPO monomers, 6 ethanol monomers or 2 turpentine oil monomers. With the proposed mapping scheme, a single chain of P123 could be represented by EO_2_PO_14_EO_2_. The mapping scheme of the CG model used in this paper is reported in Figure 1. 

According to our previous work [29], the bead-bead interaction parameters in the MD-SCF simulation method can be obtained from the Flory–Huggins χ parameters, which can be calculated by Equation (8):

(8) $$\chi_{12\;}=\frac{V_{bead}}{k_{B}T}{\left(\delta_{1}-\delta_{2}\right)}^{2}$$

where $V_{bead}$ = 600 Å^3^ in our MD-SCF simulations. The Hildebrand solubility parameter is the square root of the cohesive energy density. The cohesive energy density is the amount of energy needed to completely remove a unit volume of molecules from their neighbors to infinite separation (an ideal gas), which is equal to the heat of vaporization divided by the molar volume. 

As PEO is miscible with water at almost any concentrations, χ(PEO−Water)=0.3 was adapted [42,43,44]. In [44,45,46], corresponding to a bead volume of 150 Å^3^, the authors used the group contribution method to estimate χ(PEO−PPO)=3.0, and χ(PPO−Water)=2.1. Thus, χ(PEO−PPO)=12.0, which can be deduced based on the bead volume of 600 Å^3^. According to [47], χ(PPO−Oil)=0.9, and χ(Oil−Water)=16.8 can be deduced from their DPD simulations. Furthermore, according to [48], the solubility parameters of PEO, PPO, and ethanol are 19.9 ${(J\cdot {\mathrm{cm}}^{-3})}^{1/2}$, 18.5 ${\left(J\cdot {\mathrm{cm}}^{-3}\right)}^{1/2}$, and 26.5 ${\left(J\cdot {\mathrm{cm}}^{-3}\right)}^{1/2}$, respectively. Moreover, the solubility parameter of turpentine oil is 16.6 ${\left(J\cdot {\mathrm{cm}}^{-3}\right)}^{1/2}$, which can be obtained in the polymer handbook [49]. Thus, χ(PEO−Ethanol)=6.4, χ(PPO−Ethanol)=10.0, and χ(Oil−Ethanol)=14.3 can be calculated. Then, the interaction parameters of each component are multiplied by *RT* in the MD-SCF simulations and shown in Table 3. 

For the P123 model, the bonds between two successive beads are described by a harmonic potential:

(9) $$V_{bond}(R)=\frac{1}{2}K_{{}_{bond}}{\left(R-R_{bond}\right)}^{2}$$

where the equilibrium distance $R_{bond}$ is equal to 1.12 nm for the block copolymer. $K_{bond}=10,000\;\mathrm{kJ}\cdot {\mathrm{mol}}^{-1}\cdot {\mathrm{nm}}^{-2}$ is the bond force constant. The CG model of BCP contains no angular constraints between two consecutive bonds. All the simulations were performed by OCCAM code, which was developed by us [35]. The various analyses were computed from a single ensemble snapshot after the equilibration for each system of 2.4 µs. The calculated diffusion coefficient from the mean square displacement (MSD) of the MD-SCF simulation is $D_{polymer}=1.5\;{\mathrm{cm}}^{2}\cdot s^{-1}$ for a polymer chain of 20 beads. This value can be compared with experimental (or calculated by atomistic simulations) diffusion data from different polymers that have a similar chain size. Therefore, we can consider an order of magnitude difference in the time scaling factor~1000 from [41]. According to this, the simulations reported in our paper can be considered on the timescale of 2.4 milliseconds. 

## 3. Results and Discussion

### 3.1. Effect of Concentration on the Micelles Structure of P123 in Water

Firstly, we consider a binary system composed of P123 (with different volume concentrations) in water. For each concentration, a certain number of P123 chains were randomly placed in the box, previously filled with water CG beads (see Table 1). Then, the dilute aqueous solutions were simulated at 303K with a parallel version of the OCCAM code [35].

**Figure 2:**
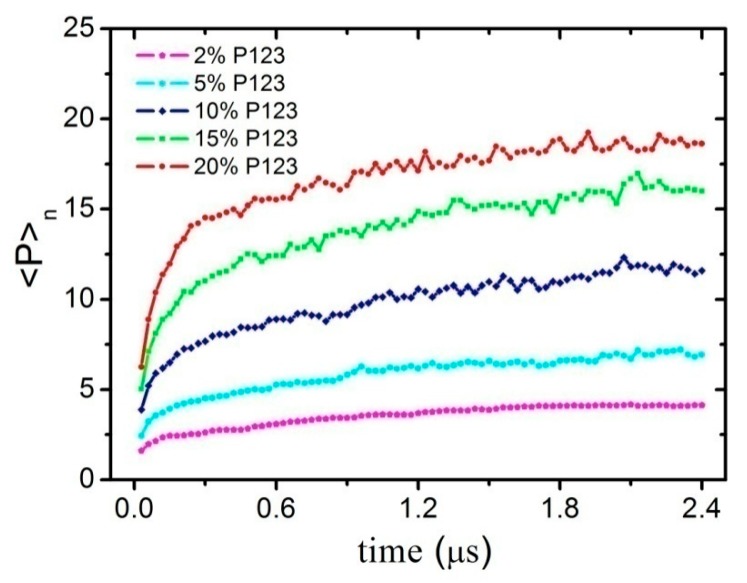
The time evolution of $<P{>}_{n}$ of micelles self-assembled by P123 dilute aqueous solution with different volume concentrations of 2%, 5%, 10%, 15%, and 20%, respectively.

For all systems, starting from approximately 200 ns, the formation of aggregates of P123 was observed. In particular, the mean aggregation number $<P{>}_{n}$ of Pluronic micelles increased with simulation time up to an equilibrium value (after 2 μs), as reported in Figure 2. In order to characterize the composition of the micelles self-assembled from P123 dilute solution, the mean aggregation number $<P{>}_{n}$ was calculated. In particular, $<P{>}_{n}$ was defined as: $<P{>}_{n}=\sum_{i}P_{i}n_{i}$, where $n_{i}$ was the fraction of the aggregates whose aggregation number equaled $P_{i}$ and $\sum_{i}n_{i}=1$ [50]. Figure 2 shows the time evolution of $<P{>}_{n}$ for micelles formed by the P123 dilute aqueous solution with different concentrations. As can be seen from Figure 2, the average composition of P123 micelles is the function of the concentration of the P123 in water. In general, the P123 micelles contain from 5 to 22 P123 chains. 

To describe the morphology of the micelles, we calculated the eigenvalues of the radius of the gyration tensor [50,51,52], as defined by

(10) $$S_{ab}=\frac{1}{n}\sum_{i=1}^{n}\left(a_{i}-a_{cm})(b_{i}-b_{cm}\right)$$

And

(11) $$\Lambda =\left[\begin{array}{ccc} S_{xx} & S_{xy} & S_{xz}\\ S_{yx} & S_{yy} & S_{yz}\\ S_{zx} & S_{zy} & S_{zz} \end{array}\right]$$

where *a* and *b* indicate *x*, *y*, or *z* components of the bead’s coordinates, respectively. Here *n* is the total number of beads in the aggregate. *a*_cm_ and *b*_cm_ are the centers of mass of the aggregate. The radius of gyration *R_g_* can be simply calculated by Equation (11):

(12) $$R_{g}{}^{2}=\lambda_{1}+\lambda_{2}+\lambda_{3}$$

where the three eigenvalues $\lambda_{1}\leq \lambda_{2}\leq \lambda_{3}$ can be obtained via diagonalization of the matrix in Equation (11). Finally, the asphericity parameter *Q* is calculated through the formula as follows:

(13) $$Q=\frac{{\left(\lambda_{2}-\lambda_{1}\right)}^{2}+{\left(\lambda_{3}-\lambda_{1}\right)}^{2}+{\left(\lambda_{3}-\lambda_{2}\right)}^{2}}{2{\left(\lambda_{1}+\lambda_{2}+\lambda_{3}\right)}^{2}}$$

**Figure 3:**
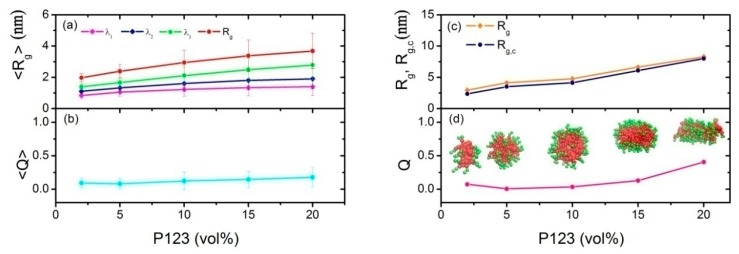
The average $\lambda_{1}$, $\lambda_{2}$, $\lambda_{3}$, and <*R_g_*> of the overall micelle (**a**), the average asphericity parameter *Q* of overall micelle (**b**), *R_g_* of the largest micelle and *R_g,c_* of the largest micellar core (**c**), and asphericity parameter *Q* of the largest micelle (**d**) corresponds to the P123 dilute aqueous solution with the volume concentration of 2%, 5%, 10%, 15%, and 20%, respectively.

In Figure 3a,b, the mean radius of gyration <$R_{g}$> and the asphericity parameter <*Q*> of the overall micelles of P123 (at the volume concentration of 2%, 5%, 10%, 15%, and 20%) are reported.

Moreover, we concentrated on the largest micelle of each system at the equilibrium state. Thus, the radius of gyration *R_g_*, the radius of gyration of the micellar core *R_g,c_*, the asphericity parameter Q, and the morphology of the largest micelle are shown in Figure 3c,d, respectively. 

In general, we observed an increase in the size of the micelles when the P123 concentration increased (see the red curve in Figure 3a), which is in agreement with the experimental behavior [13,15]. In detail, by visual inspection of simulation trajectories, we found that for a low P123 concentration, the micelles were almost spherical, while, for a higher concentration, the aggregates were much more elongated. To quantify the qualitative observation, we computed the asphericity parameter <*Q*> (in Figure 3b). We observed that for a low P123 concentration (up to 15%), <*Q*> was close to 0.5, confirming a spherical shape of the P123 aggregates, which is consistent with the experimental observations [15,53] and previous simulation studies [29,39]. This indicates that our coarse-graining procedure and our hybrid MD-SCF simulation method is appropriate for this P123 dilute solution system. From Figure 3d, we can see that *Q* equals 0.0069 and 0.035 at 5% and 10% P123 dilute aqueous solution, respectively, which means that the largest micelles at these two concentrations are closer to the regular sphere. At higher concentration of 15% and 20%, anisometric micelles can be found with *Q* values of 0.13 and 0.41, respectively. This can also be found in previous experiments [15], and is due to the larger aggregation number of P123 and larger micellar cores of the micelles. 

### 3.2. Effect of Ethanol on the Micelles Structure of P123 in the Mixed Ethanol-Water Solvent

On the basis of the coarse-graining and mesoscale simulation strategy, we then consider the self-assembly of P123 in the mixed water and ethanol solutions. The main goal of the study was to find the effect of ethanol on the micellization of Pluronic P123. To compare the simulation results with the experimental results of [20], the volume concentration of P123 was chosen by 5 % and the temperature of systems was kept at 303K. Furthermore, ethanol, at the two different concentrations of 15% and 25% was chosen, and the two different systems are shown in Table 2 (I), and (II), respectively. 

**Figure 4:**
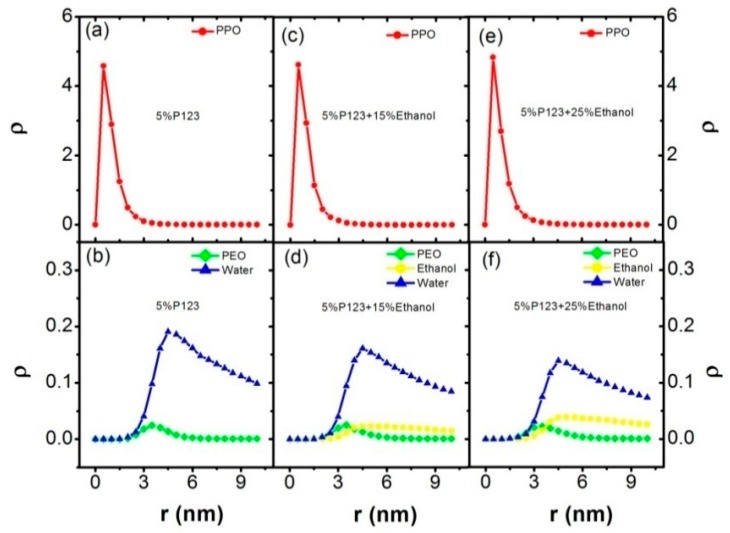
The radial density profiles (ρ) of PPO, PEO, ethanol, and water correspond to the systems of 5% P123 in water in Table 1, (I) and (II) in Table 2. (**a**) the radial density profile of block PPO in system of 5% P123 in water; (**b**) the radial density profile of block PEO and water in system of 5% P123 in water; (**c**) the radial density profile of block PPO in system of 5% P123 and 15% ethanol; (**d**) the radial density profiles of block PEO, ethanol, and water in the system of 5% P123 and 15% ethanol; (**e**) the radial density profile of block PPO in the system of 5% P123 and 25% ethanol; (**f**) the radial density profiles of block PEO, ethanol, and water in the system of 5% P123 and 25% ethanol. Block PEO: green; block PPO: red; ethanol: yellow; and turpentine oil: purple.

The radial density profiles (ρ) of PPO, PEO, ethanol, and water in these two systems were calculated in order to better describe the morphologies of the micelles self-assembled by P123 in the mixed water–ethanol dilute solution. First, we calculated the center of mass (COM) of each micelle. Then, the number density as a function of distance *r* to the COM was calculated. After that, the number density of each component was divided by the shell volume. Finally, the mean radial density profiles (ρ) were obtained by averaging the number density over all observed micelles, as shown in Figure 4. From Figure 4, we can see that the peak of block PPO (red line) corresponds to the smallest *r*, which means that block PPO chains form the core of the micelle. Moreover, the sequence of position r corresponding to the main density peaks of different components is block PPO, block PEO, ethanol, and water, respectively. This indicates that block PEO (green line) wraps block PPO as the shell of the micelle, and most ethanol is outside the micelle. Most water is mixed with the ethanol due to the similar peak location of water and ethanol. However, through comparing Figure 4b,d,f, we could find the small density distribution points formed by water (blue line) and ethanol (yellow line) were smaller than that of the block PEO, and larger than block PPO, which indicates that water and ethanol molecules are in the corona of the micelles. Furthermore, the small density peak of ethanol (yellow line) are shifted left in Figure 3f compared with Figure 3d, which means that increasing the concentration of ethanol causes ethanol molecules to move inside the PEO block. 

### 3.3. Effect of Turpentine Oil on the Micelles’ Structure of P123 in the Mixed Solvent

**Figure 5:**
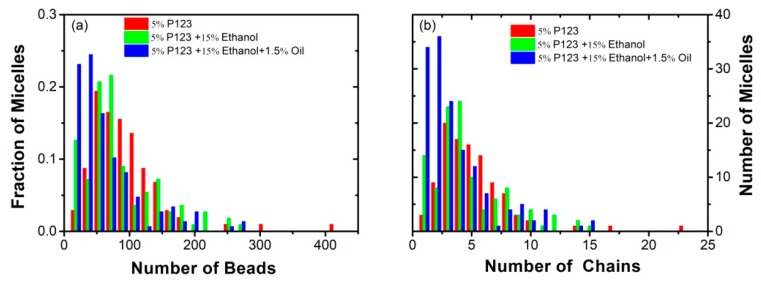
(**a**) The size distribution of the micelles corresponding to the number of beads in P123; (**b**) the number of micelles corresponding to number of chains in P123; for three systems including 5% P123 in pure water, 5% P123 in 15% ethanol and water mixed solvent (system I in Table 2), and 5% P123 in 15% ethanol, 1.5% oil, and water mixed solvent (system IV in Table 2), respectively.

To evaluate the turpentine oil effect on the micelles’ structure of P123 in the presence of ethanol, we studied three different complex systems including P123 block copolymer, ethanol, oil, and water by hybrid particle–field molecular dynamics simulation method. The system composition are reported in Table 2 (IV), (V), and (VI). In order to understand the effect of the turpentine oil on the self-assembly of micelles, the size distribution of the micelles (5% P123 in pure water, 5% P123 in 15% ethanol and water, 5% P123 in 15% ethanol, 1.5% oil, and water mixed solvent) were calculated and compared, as shown in Figure 5. Micellar Parameters of mean aggregation number <*P*>*_n_*, the mean radius of gyration<*R_g_*> and the asphericity parameter <*Q*> corresponding to the systems in Table 2 are shown in Table 4.

For the systems of 5% P123 in water, the main result that we found is a broad distribution of the micelle size. Most micelles were composed by 72 and 54 beads of P123, which corresponds to four and three chains of P123, while, the largest micelle includes 306 P123 beads (17 chains of P123). Adding 15% ethanol into the system caused a reduction of the size of micelles, and, at the same time, an increase of the fraction of micelles composed of a smaller number of P123 beads. Unlike water, ethanol is considered a good solvent for P123, as confirmed by the reduction of micelle’s radius of gyration $<R_{g}>=2.304$ nm (system (I)), compared with $<R_{g}>=4.129$ nm calculated at 5% P123 in water [18,20]. For the system (IV) of 5% P123, 15% ethanol and 1.5% oil, the percentage of free P123 chain is above 0.2. Furthermore, the mean aggregation number $<P{>}_{n}$ equals 6.095 (for the system (IV)), which is smaller than $<P{>}_{n}=6.89$ of system (I). With the introduction of the turpentine oil molecules, the main effect we observed was the increase of radius of gyration $<R_{g}>=2.462$ nm (system IV), of about 7% larger than that of system (I). This suggests that the turpentine oil molecules are mainly solubilized in the micellar core and confirms the swelling of P123 micelles [52]. 

**Figure 6:**
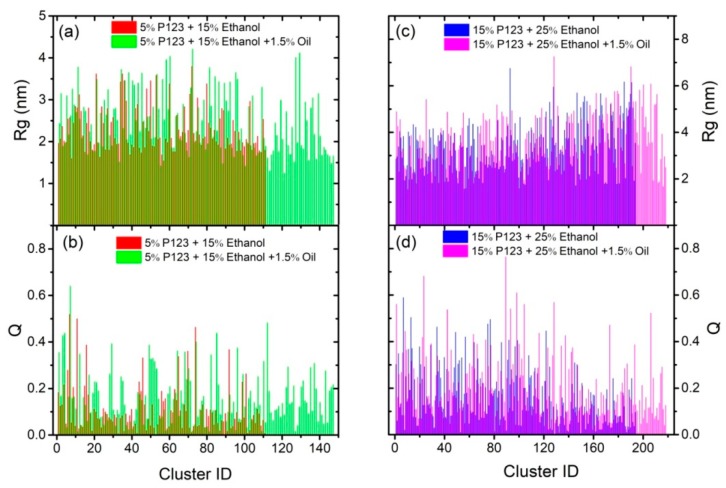
The radius of gyration *R_g_* and aspericity parameter *Q* for each cluster corresponding to the number of cluster in the systems of 5% P123 and 15% ethanol (system I in Table 2); 5% P123, 15% ethanol, and 1.5% turpentine oil (system IV in Table 2); 15% P123 and 25% ethanol (system III in Table 2); and 15% P123, 25% ethanol, and 1.5% turpentine oil (system VI in Table 2), respectively.

In order to understand the influence of turpentine oil on the P123-mediated solubilization of ethanol–water mixed solvent, we calculated the radius of gyration *R*_g_ and aspericity parameter *Q* for each cluster corresponding to each system and reported in Figure 6. Through comparing the system (I) and (IV) as shown in Figure 6a,b, a progressive increase in the number of clusters, *R*_g_, and *Q* was observed in the presence of turpentine oil (IV). The same situation was also found by increasing the concentration of P123 and ethanol, as shown in Figure 6c,d. This effect is mainly due to the swelling of micellar cores by turpentine oil molecules, were still wrapped only by PEO block molecules in the presence of oil. The number of PEO block was not enough to protect larger cores composed of PPO block and turpentine oil molecules, which prompted the formation of larger number of micelles. In the meanwhile, the spherical micelles tended to form larger cylinder micelles or wormlike micelles to obtain the PEO block’s protection, which could be observed from the larger number of *Q* > 0.5, as shown in Figure 6b,d. 

**Figure 7:**
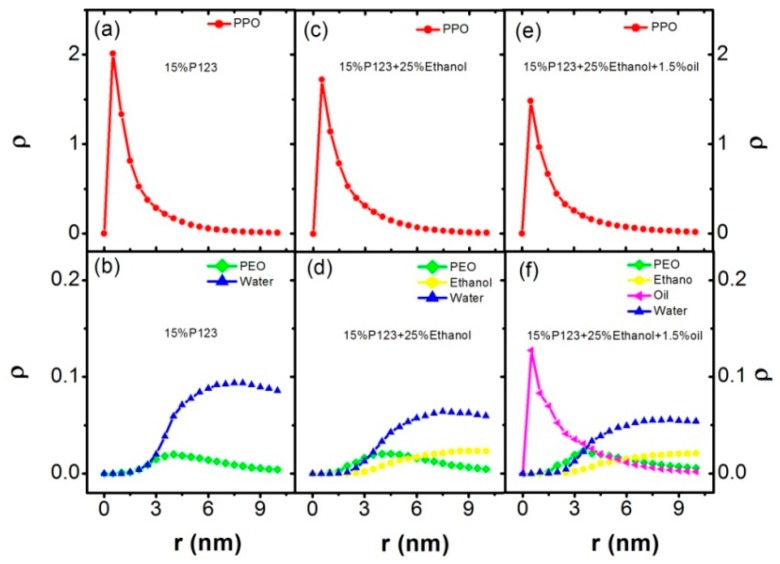
The radial density profiles (ρ) of PPO, PEO, ethanol, and water correspond to the systems of 15% P123 in water in Table 1, (III) and (VI) in Table 2. (**a**) the radial density profile of block PPO in system of 15% P123 in water; (**b**) the radial density profile of block PEO and water in system of 15% P123 in water; (**c**) the radial density profile of block PPO in system of 15% P123 and 25% ethanol; (**d**) the radial density profiles of block PEO, ethanol, and water in the system of 15% P123 and 25% ethanol; (**e**) the radial density profile of block PPO in the system of 15% P123, 25% ethanol and 1.5% turpentine oil; (**f**) the radial density profiles of block PEO, ethanol, and water in the system of 15% P123, 25% ethanol and 1.5% turpentine oil. Block PEO: green; block PPO: red; ethanol: yellow; and turpentine oil: purple.

**Figure 8:**
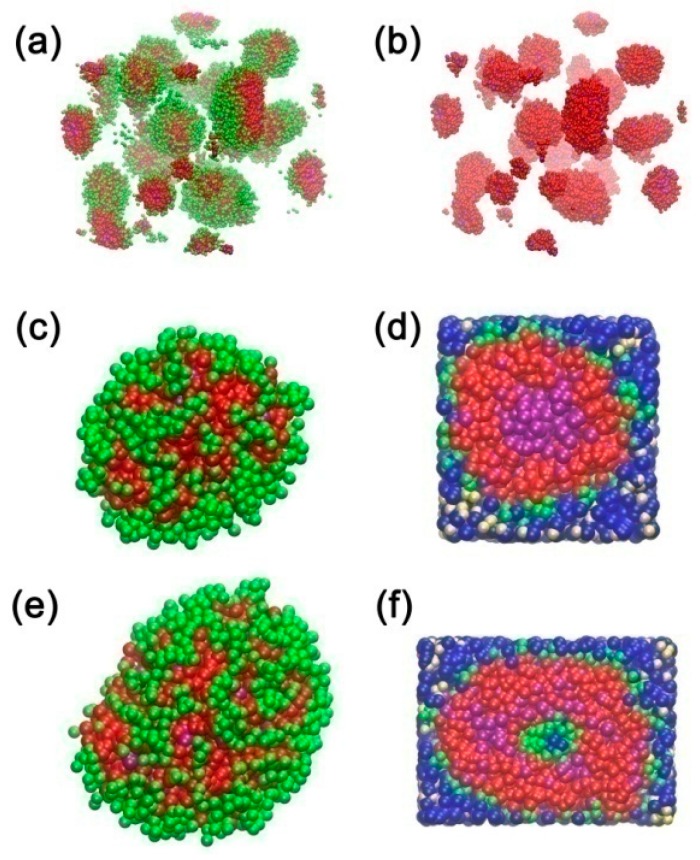
(**a**) Morphologies of micelles and vesicles in system VI (5% P123, 25% ethanol, and 1.5% turpentine oil), block PEO: green, block PPO: red, turpentine oil: purple, water and ethanol is omitted for clarity; (**b**) morphologies of cores of micelles and vesicles in system VI (5% P123, 25% ethanol, and 1.5% turpentine oil), block PPO: red, oil: purple, block PEO, water and ethanol is omitted for clarity; (**c**) morphology of whole micelle in system VI, block PEO: green, block PPO: red, turpentine oil: purple; (**d**) morphology of core of micelle surrounded by water and ethanol in system VI, block PEO: green, block PPO: red, turpentine oil: purple, ethanol: yellow, water: blue; (**e**) morphology of whole vesicle in system VI, block PEO: green, block PPO: red, turpentine oil: purple; (**f**) morphology of core of vesicle surrounded by water and ethanol in system VI, block PEO: green, block PPO: red, turpentine oil: purple, ethanol: yellow, water: blue.

The radial density profiles (ρ) of different components corresponding to 15% P123 in water, system (III) and (VI) in Table 2 were calculated and are shown in Figure 7. The main peak of density profile of the turpentine oil (purple line) shows the same position as that of block PPO, shown in Figure 7e,f, which further confirms that turpentine oil has the effect of swelling the core formed by block PPO. The morphologies of micelles and vesicles of system (VI) are shown in Figure 8. From the morphology of the micellar core of system (VI) as shown in Figure 8d, we could confirm that the turpentine oil molecules are mainly solubilized in the micellar core and block PPO chains are stretched. The same scheme is schematically presented in [20]. In the experimental work [20], they found that micellar cores are swelled significantly by the Lavender oil and results in the observed growth of the micelle. Interestingly, the morphology of vesicle and core of vesicle in system (VI) is also observed and shown in Figure 8e and f, respectively. In particular, the micelles and vesicles both exist in the systems of P123 in water/ethanol/turpentine oil mixed solvents (system (IV),(V), and (VI)).

## 4. Conclusions

We adopt a simple coarse-grained model where several segments are coarse-grained into a single bead to study the micellization of P123 in a water/ethanol/turpentine oil-mixed solvent by using the hybrid particle–field molecular dynamics (MD-SCF) method. The interaction parameters between beads of different components were estimated from Flory–Huggins parameters. Our simulations show agreement with previous experiments. For less than 20% P123 in water, the transition from sphere to cylinder micelles was observed and the micelles with larger sizes were obtained with the increase in concentration. For 5% P123 in the mixed ethanol-water solvent, when the concentration of ethanol was increased from 15% to 25%, larger sphere micelles were observed. Furthermore, in the presence of ethanol, turpentine oil could swell the core of micelles self-assembled from Pluronic P123 at the molecular level. Specifically, not only spherical micelles but also vesicles could be found in the 5% P123, 25% ethanol, and 1.5% turpentine oil dilute solution. The results in this work primarily complement previous experiments in understanding the influence of turpentine oil and ethanol on copolymer self-assembly, which will help to design effective copolymer-based formulations.

## Figures and Tables

**Table 1:** The composition of systems including block copolymer P123 and water.

Volume Fraction (vol %)	P123 No. of Atoms	Water No. of Atoms	Particle No. of Atoms	Box Length (nm)
2	3834	188,166	192,000	64.25
5	9594	182,406	192,000	64.25
10	19,206	172,794	192,000	64.25
15	28,800	163,200	192,000	64.25
20	38,394	153,606	192,000	64.25

**Table 2:** The composition of systems including block copolymer P123, water, ethanol, and turpentine oil.

System	P123 No. of Atoms	Water No. of Atoms	Ethanol No. of Atoms	Turpentine Oil No. of Atoms
I	9576 (5%)	153,624	28,800 (15%)	0
II	9576 (5%)	134,424	48,000 (25%)	0
III	28,800 (15%)	115,200	48,000 (25%)	0
IV	9576 (5%)	150,744	28,800 (15%)	2880 (1.5%)
V	9576 (5%)	131,544	48,000 (25%)	2880 (1.5%)
VI	28,800 (15%)	112,320	48,000 (25%)	2880 (1.5%)

**Table 3:** The interaction parameters χRT (kJ·mol^−1^) between different particles in the MD-SCF simulation method.

	PEO	PPO	Water	Ethanol	Turpentine Oil
PEO	0.0	30.3	0.76	16.0	21.0
PPO	30.3	0.0	21.2	25.4	2.3
Water	0.76	21.2	0.0	0.76	42.3
Ethanol	16.0	25.4	0.76	0.0	36.0
TurpentineOil	21.0	2.3	42.3	36.0	0.0

**Table 4:** Micellar Parameters of mean aggregation number <*P*>*_n_*, the mean radius of gyration <*R_g_*> and the asphericity parameter <*Q*> corresponding to the systems in Table 2.

System	Component	<*P*>*_n_*	<*R_g_*> (nm)	<*Q*>
I	5% P123 + 15% Ethanol	6.890	2.304	0.1085
II	5% P123 + 25% Ethanol	8.876	2.451	0.1216
III	15% P123 + 25% Ethanol	16.80	3.463	0.1634
IV	5% P123 + 15% Ethanol + 1.5% Oil	6.095	2.462	0.1608
V	5% P123 + 25% Ethanol + 1.5% Oil	8.558	3.038	0.1241
VI	15% P123 + 25% Ethanol + 1.5% Oil	14.25	3.544	0.1899
